# Management of hyperkalemia in the acutely ill patient

**DOI:** 10.1186/s13613-019-0509-8

**Published:** 2019-02-28

**Authors:** François Dépret, W. Frank Peacock, Kathleen D. Liu, Zubaid Rafique, Patrick Rossignol, Matthieu Legrand

**Affiliations:** 1 GH St-Louis-Lariboisière, Department of Anesthesiology and Critical Care and Burn Unit, St-Louis Hospital, Assistance Publique-Hopitaux de Paris, Paris, France; 2 University Paris Diderot, Paris, France; 3 UMR INSERM 942, Institut National de la Santé et de la Recherche Médicale (INSERM), Paris, France; 4 F-CRIN INI-CRCT Network, Vandœuvre-lès-Nancy, France; 5 Henry JN Taub Department of Emergency Medicine, Baylor College of Medicine, Houston, TX USA; 6 Department of Medicine, University of California, San Francisco, CA USA; 7 CHRU-Nancy, INSERM 1116, Université de Lorraine, CIC Plurithématique 1433, 54000 Nancy, France

**Keywords:** Hyperkalemia, Intensive care, Emergency, Renal replacement therapy, Acute kidney injury

## Abstract

**Purpose:**

To review the mechanisms of action, expected efficacy and side effects of strategies to control hyperkalemia in acutely ill patients.

**Methods:**

We searched MEDLINE and EMBASE for relevant papers published in English between Jan 1, 1938, and July 1, 2018, in accordance with the PRISMA Statement using the following terms: “hyperkalemia,” “intensive care,” “acute kidney injury,” “acute kidney failure,” “hyperkalemia treatment,” “renal replacement therapy,” “dialysis,” “sodium bicarbonate,” “emergency,” “acute.” Reports from within the past 10 years were selected preferentially, together with highly relevant older publications.

**Results:**

Hyperkalemia is a potentially life-threatening electrolyte abnormality and may cause cardiac electrophysiological disturbances in the acutely ill patient. Frequently used therapies for hyperkalemia may, however, also be associated with morbidity. Therapeutics may include the simultaneous administration of insulin and glucose (associated with frequent dysglycemic complications), β-2 agonists (associated with potential cardiac ischemia and arrhythmias), hypertonic sodium bicarbonate infusion in the acidotic patient (representing a large hypertonic sodium load) and renal replacement therapy (effective but invasive). Potassium-lowering drugs can cause rapid decrease in serum potassium level leading to cardiac hyperexcitability and rhythm disorders.

**Conclusions:**

Treatment of hyperkalemia should not only focus on the ability of specific therapies to lower serum potassium level but also on their potential side effects. Tailoring treatment to the patient condition and situation may limit the risks.

**Electronic supplementary material:**

The online version of this article (10.1186/s13613-019-0509-8) contains supplementary material, which is available to authorized users.

## Background

Hyperkalemia is a potentially life-threatening electrolyte abnormality [1–3]. Although there is no internationally agreed upon definition for hyperkalemia, the European Resuscitation Council defines hyperkalemia as a plasma level > 5.5 mmol/L and severe hyperkalemia as > 6.5 mmol/L [4]. Hyperkalemia is associated with poor outcomes in many different settings, including the acutely ill patient [5, 6]. In acute hyperkalemia, the primary mortality risks are cardiac rhythm or conduction abnormalities [7, 8]. However, the actual causes of death in patients with hyperkalemia are poorly described, and the causal relationship between hyperkalemia and outcome remains controversial.

The aim of this review is first to describe mechanisms and the risk–benefit ratio of different strategies of hyperkalemia treatment and second, to propose a tailored treatment strategy. This will include a discussion of the effectiveness as well as complications of renal replacement therapy, limiting the risk of hypoglycemia with judicious insulin and glucose administration, and the potential benefit and risks of hypertonic sodium bicarbonate.

## Methods

We searched MEDLINE and EMBASE for relevant papers published in English between Jan 1, 1938, and July 1, 2018, in accordance with the PRISMA Statement using the following terms: “hyperkalemia,” “intensive care,” “acute kidney injury,” “acute kidney failure,” “hyperkalemia treatment,” “renal replacement therapy,” “dialysis,” “sodium bicarbonate,” “emergency,” “acute.” Reports from within the past 10 years were selected preferentially together with highly relevant older publications.

### Association between hyperkalemia and outcomes

The potassium ion (K^+^) is the most abundant cation in the body. There is an estimated total reserve of 3000–4000 mmol in adults, of which only 60 mmol (2%) are extracellular [9]. Hyperkalemia is associated with poor outcomes in many different settings: in the general population [5, 6], in patients with cardiac and renal disease [10–13] and in critically ill patients [14]. In a retrospective study of hospitalized patients, Khanagavi et al. [5] found that acute kidney injury (AKI) and prolonged hyperkalemia are independent predictors of in-hospital mortality. In acute myocardial infarction, a serum potassium above 4.5 mmol/L is associated with a higher mortality [11]. More recently, Legrand et al. [15] identified that a serum potassium > 4.5 mmol/L in heart failure patients admitted to the emergency department (ED) is associated with an increased risk of death.

The net effect is a U-shaped mortality curve associated with potassium abnormalities [16–19]. Several observational studies have identified hypokalemia as an independent risk factor for poor outcome [13, 16–19]. This association raises concern regarding the potential for overcorrection, as may occur with some fast-acting potassium-lowering agents. However, these associations do not mean causality and thresholds for treating hyperkalemia remain debated.

### Cardiac manifestations of hyperkalaemia

Although patients with hyperkalemia can present rarely with weakness progressing to flaccid paralysis, paresthesias, or depressed deep tendon reflexes, the clinical presentation of hyperkalemia is usually benign until cardiac rhythm or conduction disorders occur. Elevation of extracellular potassium has several effects on myocardial electrophysiology that contribute to intracardiac conduction disturbances. The intracellular to extracellular potassium gradient lessens when extracellular potassium increases, thus decreasing the resting membrane potential. Elevated extracellular potassium also increases membrane permeability for potassium, lowers membrane resistance, increases repolarizing currents, and shortens transmembrane action potential duration.

While rising serum potassium initially increases conduction velocity, it decreases it at higher levels [20]. Classic hyperkalemia electrocardiographic findings include signs of hyperexcitability such as peaked T-waves (reflecting a decrease in the threshold for rapid depolarization). Further, altered conduction may manifest as PR prolongation, loss of P-waves, QRS widening, bradycardia, and ultimately a sine wave rhythm due to action potential shortening and prolongation of diastolic depolarization.

**Fig. 1:**
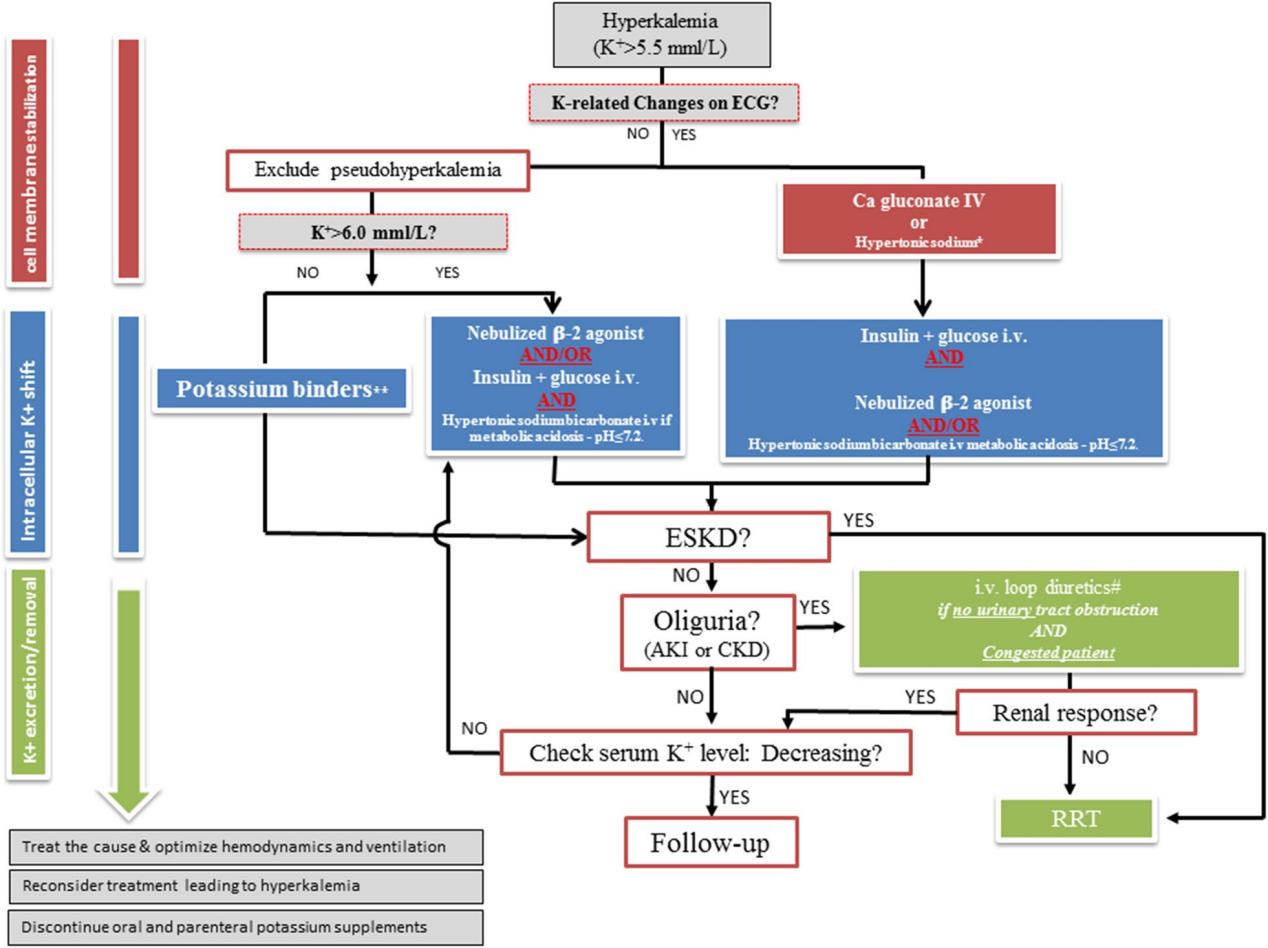
Suggested algorithm for hyperkalemia treatment in the acutely ill. *In case of Digitalis intoxication or hypercalcemia. **Sodium zirconium cyclosilicate and patiromer when available, kayexalate if not available. *ESKD* end-stage kidney disease, *AKI* acute kidney injury, *CKD* chronic kidney disease, *RRT* renal replacement therapy

Importantly, the correlation between potassium elevation and electrocardiographic (ECG) changes is poor. Severe hyperkalemia may manifest with minimal or atypical ECG findings [1–3, 21], including nonspecific ST segment modification or pseudo-Brugada syndrome (featuring wide QRS, elevation of the ST segment, J-point elevation, T-wave inversion). On the contrary moderate hyperkalemia (< 6 mmol/L) may have life-threatening ECG findings. The electrocardiographic manifestations of hyperkalemia are largely influenced by rapid changes of plasma concentration [7], the gradient of potassium across the myocardial cell membrane, the effect of other ions (i.e., sodium, calcium), as well as underlying cardiac disease [22]. Retrospective data found a higher mortality rate in patients with hyperkalemia showing abnormal ECG findings [23]. Along these lines, chronically dialyzed patients may show no ECG signs of hyperkalemia despite high serum potassium levels. Altogether, more than the absolute serum potassium level, therapeutic strategies should be guided by the cardiac consequences of hyperkalemia identified on the ECG (Fig. 1).

### Causes of hyperkalemia in acutely ill patients

Factors associated with the development of hyperkalemia can be classified into three categories, and include altered renal clearance of potassium (e.g., chronic kidney disease, acute kidney injury, renin–angiotensin–aldosterone system inhibitor), release from the intracellular space (e.g., hemolysis, rhabdomyolysis, tissue injury) and altered transfer to the intracellular space (e.g., acidosis, insulin deficit, β-adrenergic blockers, heparin) (Table 1). Hyperkalemia in the patient with normal renal function is unusual and should prompt evaluation for pseudo-hyperkalemia if no ECG abnormalities consistent with hyperkalemia are identified (false elevation of potassium due to hemolysis occurring with blood draw and not reflective of the patient’s plasma potassium concentration). While concomitant medications (e.g., potassium supplements, penicillin G, digoxin, nonsteroidal anti-inflammatory drugs, renin–angiotensin–aldosterone system inhibitor, amiloride, triamterene, trimethoprim, pentamidine) are often a contributor to hyperkalemia, in our experience they are rarely the only cause in acute settings.

**Table 1:** Mechanisms contributing to the development of hyperkalemia

Mechanisms contributing to the development of hyperkalemia	
Increased extracellular K^+^	Decreased K^+^ elimination
Tissue injury Hemolysis Rhabdomyolysis Tumor lysis syndrome K^+^ shift in extracellular space Mineral acidosis (i.e., hyperchoride acidosis) Succinylcholine Inability to enter into myocyte Diabetes mellitus Hyperglycemia Hypertonicity β_2_-receptor antagonists Aldosterone blockers Cardiac glycosides High acute iatrogenic K^+^ load Increased dietary intake Blood transfusion Error of injection	AKI Hypovolemia Sepsis Acidosis treatment RAAS inhibitor Calcineurine inhibitor Cardiac glycosides

*K*^*+*^ potassium, *RAAS* renin–angiotensin–aldosterone system

Since the potassium pool is mostly intracellular, alteration of cellular potassium uptake can be a major contributors to hyperkalemia [24]. Hyperchloremic acidosis is frequent in acutely ill patients [25]. According to the Stewart’s theory, the main determinant of acid–base balance is the strong ion difference (SID), essentially determined by the difference between the strong cation (sodium) and the anions (chloride) [26]. A possible mechanism to explain hyperkalemia related to hyperchloremic acidosis is that mineral acids (i.e., chloric) cannot freely diffuse into the intracellular compartment, they decrease extracellular pH. Low extracellular pH decreases the Na^+^–H^+^ exchange and inhibits the inward movement of Na^+^. The subsequent fall in intracellular Na^+^ reduces Na^+^–K^+^-ATPase activity, leading to a net decrease in K^+^ transfer into the cell and higher extracellular potassium levels. In this line, utilization of balanced solutions with physiological concentrations of chloride (i.e., Ringers lactate) prevents the development of mineral metabolic acidosis and is associated with lower serum potassium levels compared to NaCl 0.9% [25, 27, 28]. The effect of metabolic acidosis appears less prominent when organic acids accumulate (i.e., lactate, phosphate). This is because organic acids can passively diffuse into the intracellular compartment, resulting in a larger fall in intracellular pH. The fall of intracellular pH stimulates inward Na^+^ movement and maintains Na^+^–K^+^-ATPase activity, which minimizes the extracellular accumulation of potassium [29]. Ultimately, the increased intracellular Na^+^ concentration leads to the intracellular entry of potassium [29].

A special warning should be made with regards to the use of succinylcholine, classically used to induce paralysis in acutely ill patients for rapid sequence intubation. Succinylcholine induces skeletal muscle cell depolarization with an efflux of intracellular potassium by nicotinic receptor activation. In a population of critically ill patients, succinylcholine increased serum potassium on average 0.4 mmol/L (interquartile range 0–0.7 mmol/L) [30]. It should be avoided in patients with hyperkalemia and in patients with up-regulation of nicotinic receptors, as they are at risk of greater potassium elevation. This includes those with anatomical denervation, prolonged administration of neuromuscular blocking drugs, burn injury, and prolonged immobilization [31]. Alternative to succinylcholine are available in patients at risk of hyperkalemia (i.e., rocuronium).

## Medical strategy

### First-line treatment in hyperkalemia with ECG abnormalities: myocardial protection

#### Calcium salt

**Fig. 2:**
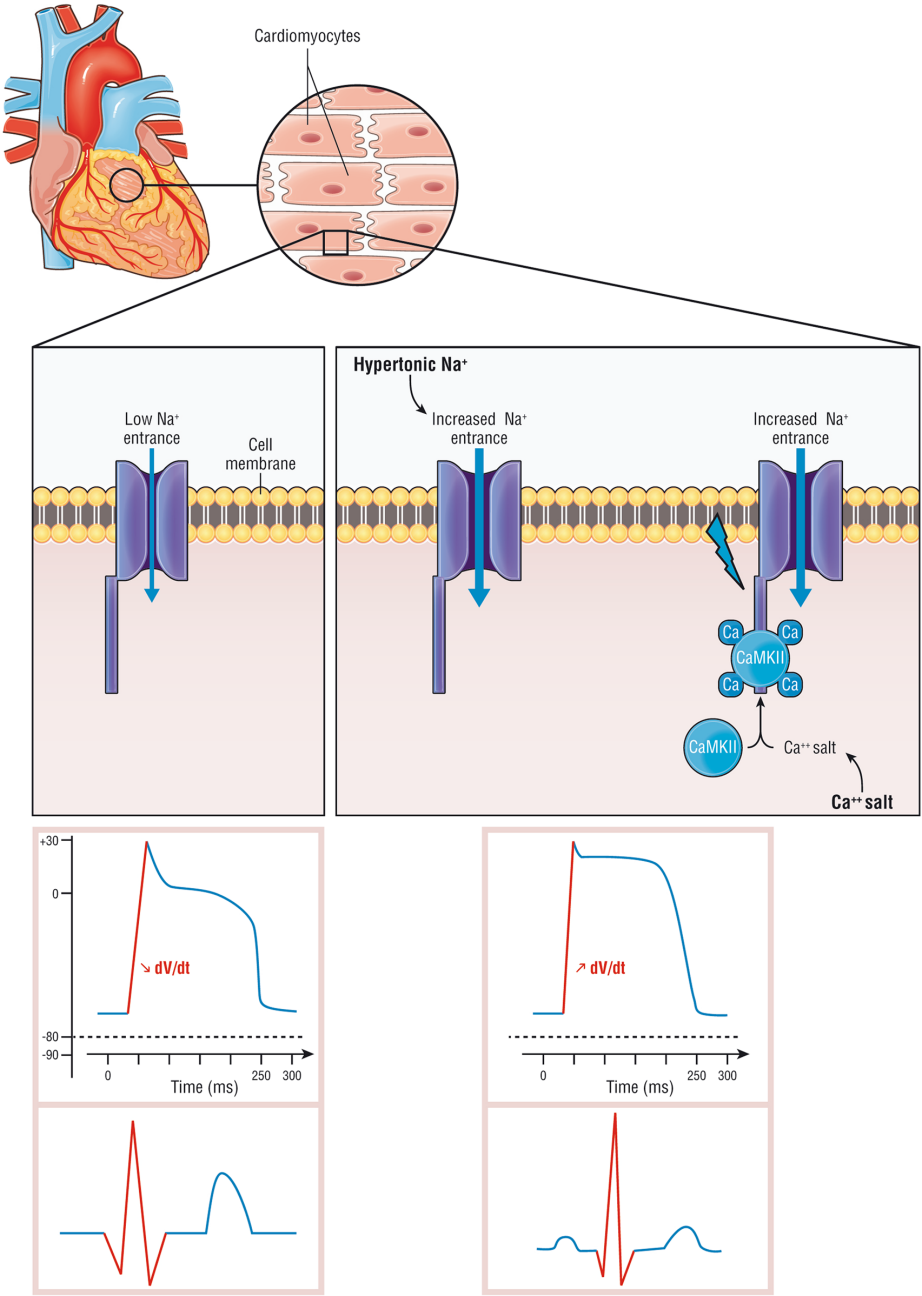
Cardiac effect of hypertonic sodium and calcium salt during hyperkalemia. During hyperkalemia, resting membrane potential increases, derecruiting the sodium voltage gate channel Nav1.5 (left panel). Calcium salts bind to calcium-dependent calmodulin and protein kinase II (CaMKII) and activates the sodium voltage gate channel leading to an intracellular sodium entrance (right panel). Calcium salt restores the channel activity though the calcium-dependent calmodulin (CaM), recruiting the voltage-gated channel Nav1.5, increasing the intracellular sodium entrance, restore dV/dt phase 0 action potential and increase in the resting membrane potential. Hypertonic sodium increases extracellular sodium concentration and “forces” intracellular sodium entrance (right panel). The bottom panel represents on the left the decrease of dV/dt phase 0 action potential due to hyperkalemia (Bottom left panel), restored by either calcium or hypertonic sodium (Bottom right panel)(Adapted from [40, 41] with authorization)

The intravenous administration of a calcium salt increases the cardiac threshold potential, the speed of impulse propagation and stabilizes the myocellular membrane, thus causing almost immediate normalization of the ECG abnormalities (Fig. 2). In 1950, Merrill et al. [32] found a beneficial effect of intravenous calcium salt in 9 of 10 patients with hyperkalemia. Four years later, this was confirmed by Chamberlain et al. [33], who reported five cases of an immediate effect of intravenous calcium on ECG changes induced by severe hyperkalemia (from 8.6 to 10 mmol/L). There are no randomized studies to show its efficacy and its indications are based on expert opinion [34]. The effect should be immediate (within 5 min) when any hyperkalemia-related ECG changes are identified or suspected [33]. The protective effect may last between 30 and 60 min [35]. Calcium administration in the case of hypercalcemia may be problematic. It also increased toxicity with digoxin overdose in animal models [34]. However, this effect was found only at nonphysiologically high calcium concentrations [35]. The use of calcium in cases of hyperkalemia associated with digoxin toxicity was not associated with life-threatening dysrhythmias or mortality in human studies [36–38]. Finally, calcium may cause tissue injury (i.e., skin necrosis) in case of extravasation [39]. The recommended dose is 10–20 mL of a 10% calcium salt (e.g., 1–2 g of gluconate or chloride).

### Hypertonic sodium

Infusion of hypertonic sodium also increases the action potential rising velocity in isolated cardiomyocytes [42]. In 1960, Greenstein et al. [43] studied the effect of sodium lactate, sodium bicarbonate, and sodium chloride on ECG abnormalities induced by hyperkalemia in nephrectomized dogs. Infusion of hypertonic sodium increased the action potential rising velocity, which was depressed when isolated cardiomyocytes were exposed to increasing concentrations of potassium [42]. Taken together, these results suggest that hypertonic sodium acts as a membrane stabilizer and might be considered as an alternative to calcium in hyperkalemia-induced ECG changes when infusion of calcium is at risk. Furthermore, the fluid loading associated with hypertonic sodium bicarbonate may increase the glomerular filtration rate and renal potassium excretion in volume-depleted patients.

### Intracellular potassium transfer

#### Hypertonic sodium bicarbonate

Although the data supporting the use of sodium bicarbonate as a treatment for hyperkalemia are controversial, it does have effects on serum potassium after infusion of hypertonic sodium bicarbonate. Some reported little effect on the potassium concentration in stable hemodialysis patients [44, 45]. In 1997, Ngugi et al. [46] observed that bicarbonate was less effective than salbutamol and insulin–dextrose in groups of 10 patients with end-stage renal disease (i.e., not acutely ill). Others reported effects on serum potassium. Schwarz et al. [47] reported that an infusion of 144–408 mmol of sodium bicarbonate over 2–4 h lowered the serum potassium by 2–3 mmol/L in four patients with severe acidosis.

In a recent randomized controlled trial (RCT), hypertonic sodium bicarbonate (4.2%) was administered to critically ill patients with severe metabolic acidaemia (pH < 7.2) [48]. There was no difference in the primary outcome (composite of death from any cause by day 28 or 1 organ failure at day 7), but the sodium bicarbonate group had significantly lower potassium concentrations compared to the control group and required renal replacement therapy less frequently. A more recent retrospective study also reported improved survival in septic patients with AKI stage 2 or 3 and severe acidosis treated with sodium bicarbonates infusion [49]. However, the impact on serum potassium was not reported.

Metabolic alkalosis, hypernatremia, hypocalcemia, and fluid overload are potential expected side effects of sodium bicarbonate (Table 2). Hypertonic sodium bicarbonate can cause hypocalcaemia in a pH dependent manner and by direct calcium binding [50]. In the Jaber et al. [48] study, more patients in the bicarbonate group had ionized calcium lower than 0.9 mmol/L compared to patients in the placebo group (24% vs 15%, *p* = 0.0167) and 2 patients had a ionized calcium below 0.5 mmol/L in the bicarbonates group versus none in the placebo group. Calcium is key for cardiac contractility. In an experimental model of lactic acidosis, Kimmoun et al. [51] reported improved myocardial elastance, aortic and mesenteric vasoreactivity when sodium bicarbonate was combined with calcium compared to sodium bicarbonate alone. Severe hypocalcemia can cause myocardial dysfunction and therefore ionized calcium should be monitored and ionized hypocalcemia corrected after sodium bicarbonate infusion. Finally, even though sodium bicarbonate has been suspected of causing intracellular acidosis, this has not been confirmed in vivo [52]. We therefore recommend to use hypertonic sodium bicarbonate (e.g., 100–250 mL of 8.4% sodium bicarbonate over 20 min) in patients with metabolic acidosis (pH < 7.2) or in patients with a contraindication to calcium administration (patients with hypercalcemia and/or severe digoxin intoxication), whether sodium bicarbonate is efficient in reducing serum potassium in patients without severe acidosis and the impact of the mechanism of metabolic acidosis need further exploration.

**Table 2:** Treatments of hyperkalemia

Type of treatment	Effect on potassium plasma level	Administration	Potential side effects	Population at risk	Preferred population
*Myocardial protection*					
Calcium salt	None	10–20 mL of calcium gluconate 10% i.v within 5 min	Hypercalcemia	Digitalis intoxication or hypercalcemia	Hyperkalemia with ECG modifications
Hypertonic sodium (e.g., sodium bicarbonate)	− 0.47 ± 0.31 mmol/L at 30 min	10–20 mL of sodium chloride 20% i.v within 5 min or 100 mL of 8.4% i.v sodium bicarbonate	Venous toxicity, increasing PaCO_2_ (due to bicarbonate)	Hypervolemia, patients with heart failure, hypernatremia, patient with respiratory insufficiency (due to bicarbonate)	Hyperkalemia with ECG modifications, patient with metabolic acidosis or AKI
*Intracellular potassium transfer*					
Insulin dextrose	− 0.79 ± 0.25 mmol/L at 60 min	5 UI of rapid insulin + 25 grams of dextrose over 30 min or 10 of rapid insulin + g of dextrose or 0.5 U/kg of body weight	Hyperglycemia and hypoglycemia	All patients	Severe hyperkalemia with hourly monitoring of plasma glucose possible
				Critically ill patients at increased of hyperglycemia-related side effects	
				Patients with acute neurological disease	
β2 mimetics	− 0.5 ± 0.1 mmol/L at 60 min	10 mg nebulized salbutamol	Tachycardia, arrhythmias, myocardial ischemia	Patients with ischemic cardiopathy	Patient without heart failure, angina or coronary disease
			Increase plasma lactate level	Patient under β blockers therapy	Spontaneously breathing patient
*Elimination*					
Renal replacement therapy	− 1 mmol/L within minutes	High blood flow and dialysate flow in hemodialysis, high ultrafiltration rate in hemofiltration	Complications related to catheter (i.e., infection, thrombosis, hemorrhage)	Low availability of the technique	Severe renal failure, multiple organ failure
				Delay to initiate the treatment	
Loop diuretics	Unpredictable	Variable	Hypovolemia, hypokalemia, hypomagnesemia	Hypovolemic patients	Hypervolemic patients with normal or moderately altered renal function
*Absorption*					
Sodium polystyrene sulfonate	Unpredictable (no randomized controlled trial in acute hyperkalemia)	15 g one to four times per day	Digestive perforation, hypocalcemia, hypomagnesemia	Patients with abnormal transit, critically ill patients	Treatment of chronic hyperkalemia
Patiromer	0.21 ± 0.07 mmol/L within 7 h (no randomized controlled trial in acute hyperkalemia)	8.4–25.2 g per day	Potential interaction with co-administered drugs, hypomagnesemia, potential long-term calcium disorder	Patients with abnormal transit	Treatment of chronic hyperkalemia
ZS-9	0.6 ± 0.2 mmol/L within 2 h	10 g one to three times per day	Edema	Patients with abnormal transit	Treatment of chronic and potentially acute hyperkalemia

*i.v* intravenous, *ECG* electrocardiographic, *β2* beta 2, *ZS-9* sodium zirconium cyclosilicate

#### Insulin–dextrose

**Fig. 3:**
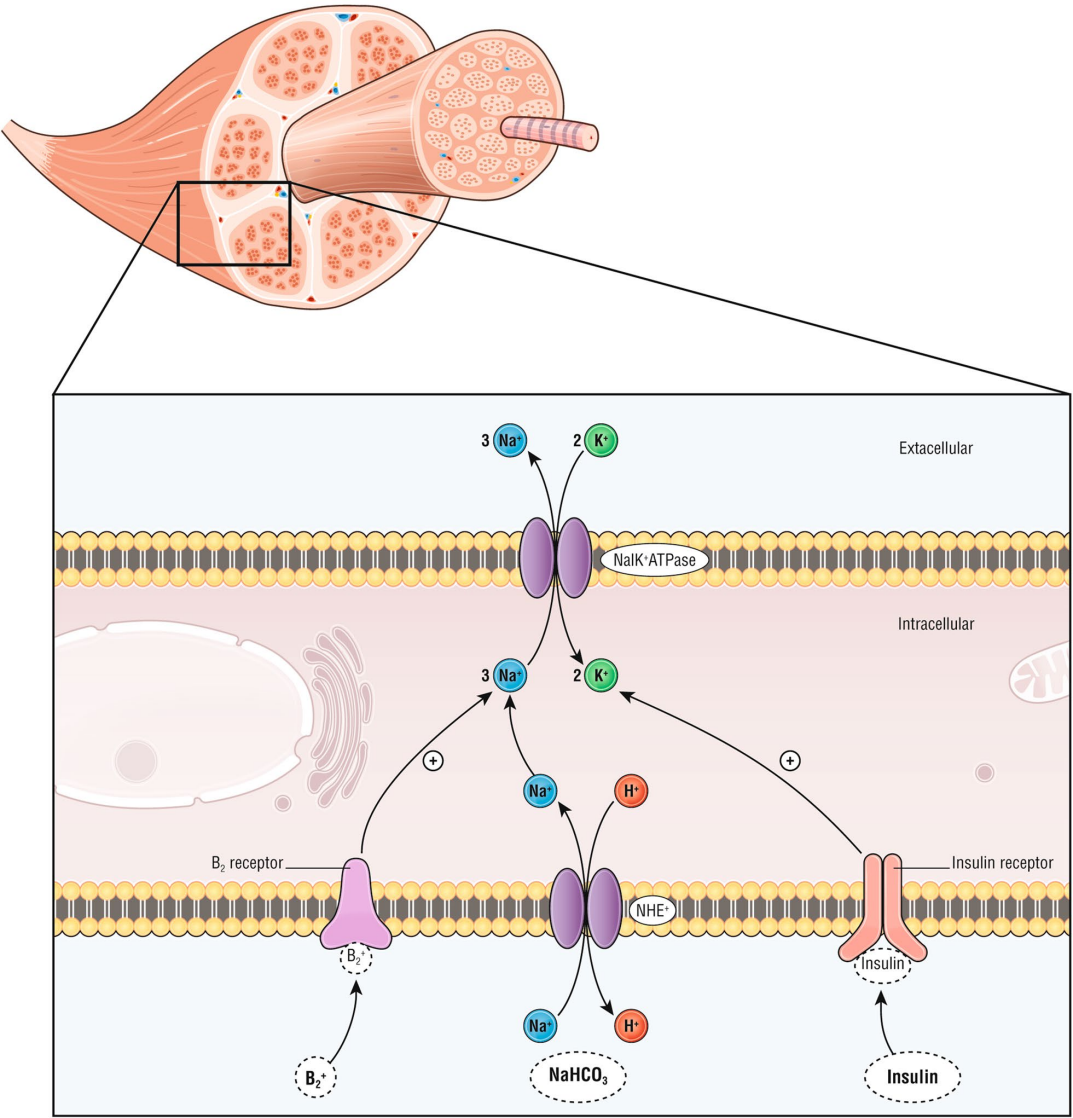
Action mechanisms of plasma lowering treatments by intracellular transfer. β-2 agonist (i.e., salbutamol) binds the β-2 receptor, insulin binds insulin receptors and sodium bicarbonate (NaHCO_3_) induces an intracellular entrance of sodium through the Na^+^/H^+^ exchanger (NHE), all activate the sodium–potassium adenosine triphosphatase (NaK^+^ ATPase) leading to a potassium transfer from the extracellular space to the intracellular space

Insulin binds to the insulin receptor on skeletal muscle, activates the sodium–potassium adenosine triphosphatase, and leads to potassium transfer from the extracellular to intracellular space (Fig. 3) [53]. Although insulin–dextrose has never been tested versus placebo for the treatment of hyperkalemia, it shows similar effects on serum potassium compared with salbutamol in a study of 20 patients [46, 54] but with faster decrease in serum potassium with insulin (i.e., 15 vs 30 min). Of note, combination of both further lowered serum potassium compared to separate treatments. The major side effect of insulin is hypoglycemia, which has been reported to occur up to 75% in subjects, depending of the protocol [55, 56]. One of the few blinded ED studies of hyperkalemia management found a 17% rate of clinically significant hypoglycemia after insulin–dextrose therapy [53].

Several studies suggest that a lower bolus dose of insulin may be safer. In 2 retrospective studies, similar potassium-lowering effects were found with the administration of either 5 or 10 U of insulin (and 25 g of dextrose), but a higher incidence of hypoglycemia occurred with the higher insulin dose [57, 58]. To limit hypoglycemia with the 10 U insulin dose required using 50 g to 60 g of dextrose [59]. Another strategy is to administer weight-based insulin dosing (0.1 U/kg of body weight up to a maximum of 10 U) to limit episodes of hypoglycemia without impacting potassium lowering [60]. Finally, using an infusion limited to 30 min led to a faster decrease in potassium, but less hypoglycemia as compared to continuous infusion [61]. Ultimately, because of the risk of hypoglycemia, blood glucose should be measured on an hourly basis for at least 2 h, and potentially longer in the setting of renal failure [61]. While the risks of hypoglycemia have long been recognized, the risk of hyperglycemia is probably underappreciated. To summarize, using 5 U of insulin with 25 g of dextrose appears an effective and safe regimen. The impact of exogenous administration of insulin and glucose on serum potassium and organ damage in this setting is unknown. Intravenous administration of high doses of glucose to limit the risk of hypoglycemia may induce severe hyperglycemia, which has been associated with organ damage, vascular dysfunction and poor outcomes in different settings (i.e., heart failure, sepsis, critically ill patients) [62–64]. Critically ill patients often present with hyperglycemia and insulin resistance. We propose insulin–glucose as first-line treatment in patients with relative contraindication to β-2 agonists (Table 2) and patients with severe hyperkalemia (i.e.,  ≥ 6.0 mmol/L or associated with ECG changes).

### β-2 agonists

Salbutamol (e.g., albuterol) is effective at lowering potassium, without differences between nebulized or intravenous administration, in terms of its efficacy [65, 66] even though effectiveness appears variable. However, salbutamol administered intravenously is associated with more cardiovascular side effects than the nebulized route [67]. In one study of 10 patients treated with 10–20 mg salbutamol, the maximal decrease in potassium ranged from 0.4 to 1.22 mmol/L [65, 66]. The peak effect occurred between 60 and 90 min after administration, and the higher salbutamol dose was more efficient in lowering potassium. Due to systemic effects of salbutamol, regardless of the route of administration, side effects, such as tachycardia may also be of concern in patients with heart failure or unstable angina. Finally, other consequences of β-2-agonists are hyperglycemia and increased plasma lactate. Impacts of treatments with β-blockers or efficacy in critically ill patients remain unexplored. Critically ill patients may present sympatho-adrenal activation (i.e., with tachycardia, vasoconstriction, hyperglycemia). We recommend the utilization of 10 mg nebulized salbutamol as first-line therapy in nonsevere hyperkalemia in spontaneous breathing patients without tachycardia.

### Increase potassium urinary excretion

Loop diuretics inhibit the NKCC2 channel at the apical surface of thick ascending limb cells along the loop of Henle. NKCC2 is a sodium–potassium–chloride cotransporter that reabsorbs (directly and indirectly) up to 25% of filtered sodium and chloride. Its blockade is responsible for most natriuretic effects of loop diuretics [68]. Loop diuretic administration via the intravenous route is quickly followed by a similar dose dependent increase in both 24-h kaliuresis and natriuresis [69, 70]. The kaliuretic effect is predominately a function of an increased tubular flow rate and a higher sodium concentration in the late nephron, both leading to an induction of the Na/K^+^-ATPase that increases potassium excretion in the distal tubules and collecting duct [70]. However, one major drawback of diuretics is the unpredictable natriuretic and kaliuretic effects, especially in patients with AKI or heart failure. These patients may be resistant to the diuretic and kaliuretic effects of diuretics, thus making this a poor strategy to control severe hyperkalemia. A “furosemide stress test” has been proposed in AKI patients to predict sustained AKI, with nonresponders defined as a urine output < 200 mL in the first 2 h after an infusion of 1.0 or 1.5 mg/kg of furosemide [71]. In these nonresponders, alternative strategies to control hyperkalemia should not be delayed. Furthermore, loop diuretics should be titrated (0.2–0.4 mg/kg in patient without AKI to 1–1.5 mg/kg of furosemide in patients with AKI) and only considered in patients with fluid overload after excluding low intravascular volume and with close attention to the amount of diuresis to avoid additional kidney insults resulting from iatrogenic hypovolemia. Finally, close monitoring for potential side effects, including the risk of secondary hypovolemia and other electrolytes disturbances (i.e., dysnatremia, metabolic alkalosis, hypophosphatemia, hypomagnesaemia) is needed. To conclude, except in patients with symptomatic fluid overload, diuretics should not be considered as a therapy for hyperkalemia.

### Gastro intestinal excretion

#### Sodium polystyrene sulfonate (SPS)

SPS exchanges sodium for calcium, ammonium, and magnesium in addition to potassium in the colon (Additional file 1: Figure S1) [72]. To date, no controlled trials in humans or animals have demonstrated that SPS increases fecal potassium losses, and no studies on the efficacy of SPS are available in the acute setting. However, serious gastrointestinal complications related to SPS, and attributed to sorbitol (co-administered with SPS to increase its delivery to the colon) have been described [73]. These include intestinal perforations, especially in patients with abnormal transit (e.g., patients in shock or who are immediately postoperative). Furthermore, its use has been associated with edema and increases in blood pressure-likely related to the fact that it exchanges potassium for sodium. Due to its route of administration, its delayed and highly variable onset, and the potential for serious adverse side effects [35, 73], SPS is not a treatment of choice in the acutely ill patient.

### Emerging treatment alternatives

#### Patiromer

Patiromer is a sodium-free, nonabsorbed, potassium-binding polymer, approved in the USAUS and in the European union (EU) for management of hyperkalemia. In a recent meta-analysis of phase 2 and phase 3 trials, it was associated with a decrease in serum potassium of 0.21 ± 0.07 mmol/L within 7 h [74, 75]. Its long term efficacy and safety was also shown in a 52-week trial [76]. Side effects include minor gastrointestinal intolerance and hypomagnesemia (7.1%) and edema due to exchange of potassium for sodium [75]. Patiromer has not been clinically tested in the emergency setting. Whether this compound may enable the maintenance of normokalemia in emergency room patients is currently being tested (REDUCE study NCT: 02933450).

#### Sodium zirconium cyclosilicate (ZS-9)

ZS-9 is a crystal that is highly selective for potassium and ammonium ions exchanging sodium for potassium [77]. A recent meta-analysis of phase 2 and phase 3 studies concluded that ZS-9 was effective in maintaining normokalemia with minor gastrointestinal side effects and edema [75]. Even though ZS-9 has not been specifically compared to existing alternatives for treatment of severe hyperkalemia in emergency conditions, Kosiborod et al. [78] recently described a subgroup of 45 patients with severe hyperkalemia (> 6 mmol/L) who received a 10 g dose of ZS-9. The median time to a serum potassium level < 6.0 mmol/L was 1.1 h, and the median time to a level ≤ 5.5 mmol/L was 4.0 h, suggesting that this treatment might be considered in severe acute hyperkalemia in patients with preserved gastrointestinal function. However, because of the lack of data in the acute setting and its potential delayed onset of action, it was not approved for acute hyperkalemia management in either the US or in UE. An ongoing phase 2 study (NCT03337477) is evaluating the short term efficiency of ZS-9 plus insulin–dextrose versus insulin–dextrose alone in patients with acute hyperkalemia.

### Renal replacement therapy

#### Indication of Renal replacement therapy

Severe hyperkalaemia is a key indication for renal replacement therapy (RRT) (e.g., hemodialysis or hemofiltration) in acutely ill patients with AKI [8]. However, what potassium concentration or other clinical indications (e.g., significant ECG changes) should serve as triggers for RRT remain debated [8]. However, the literature does however provide some guidance [79]. In a recent trial, a strategy of delayed RRT (with timing of RRT determined by serum creatinine or urine output) ultimately avoided RRT in many patients [80]. Not unexpectedly, medical treatment for hyperkalemia was more frequent in the delayed group, but the incidence of arrhythmias did not differ between groups. Of note, patients with potassium > 6, or > 5.5 mmol/L despite medical treatment, were excluded, a factor limiting conclusions regarding acute therapy in those with the most severe hyperkalemia. Another trial evaluated hypertonic sodium bicarbonate in critically ill patients with severe acidaemia (pH < 7.2). They reported the bicarbonate group had a lower serum potassium, less need for RRT, and a longer delay to RRT in those patients ultimately requiring RRT [48]. Altogether these data suggest that medical treatment of hyperkalemia (including hypertonic sodium bicarbonate in patients with metabolic acidosis) may be safe in critically ill patients with mild hyperkalemia. This medical treatment could avoid or delay RRT onset in patients with AKI.

#### Renal replacement therapy and potassium dialysance

**Fig. 4:**
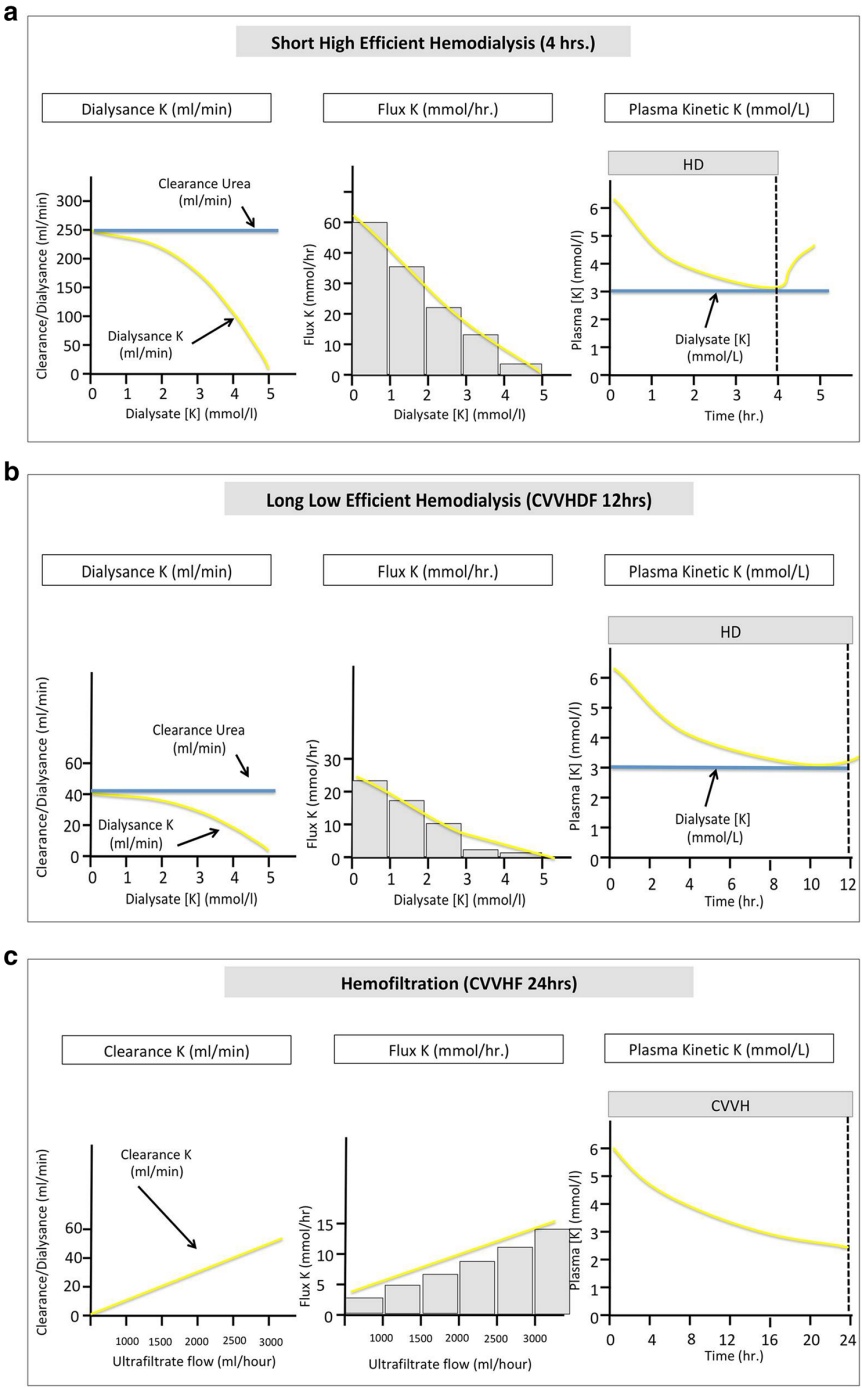
Action mechanisms of hypokalemic treatments by intracellular transfer. **a** Potassium dialysance, flux and plasma kinetic under short high efficient hemodilaysis. **b** Potassium dialysance, flux and plasma kinetic under long low efficient hemodilaysis. **c** Potassium clearance, flux and plasma kinetic under hemofiltration. *K* potassium, *CVVHD* continuous venovenous hemodialysis, *CVVHF* continuous venovenous hemofiltration

Renal replacement therapies (RRT) include diffusive (i.e., hemodialysis), convective (i.e., hemofiltration) and mixed modalities (e.g., hemodiafiltration) in the acute setting. Potassium dialysance refers to the clearance of potassium in various RRT modalities. Body potassium dialysance and potassium flux depends on the gradient of potassium concentration between plasma and dialysate (or infusate using hemofiltration), blood and dialysate flow through the circuit, the modality (hemodialysis, hemofiltration, hemodiafiltration), and the dialyzer characteristics. Potassium mass transfer on the other side depends on treatment time and intracorporeal potassium kinetics (Fig. 4). Since potassium freely and totally diffuses throughout the dialyzer membrane, it is rapidly and effectively removed during hemodialysis. In the setting of high blood and dialysate flow and low dialysate potassium concentration, serum potassium drops within minutes of initiation. Since intracorporeal potassium kinetics behave as a multi-compartmental model, serum potassium will decrease more slowly after 2 h of hemodialysis and rebound after stopping the therapy. Of note, hyperosmolarity, or treatments shifting potassium from the extracellular to the intracellular space before the dialysis session (i.e., β-2 agonists, sodium bicarbonate, insulin, glucose), will decrease potassium dialysance.

Continuous RRT, including hemofiltration (i.e., convective technique), is the most frequently used modality in the intensive care unit. Using convective techniques, flux of potassium through the membrane depends on the ultrafiltration rate and the serum potassium level (Fig. 4). When combined techniques are used (i.e., hemodiafiltration), elimination of potassium depends mostly on the diffusive transfer through the membrane. Continuous low flow techniques have a slower decrease in serum potassium concentrations, and the serum potassium will tend to approach dialysate (with diffuse techniques) or infusate concentration (with convective techniques) within few hours after initiation without rebound. Hemofiltration using mild to high cut-off membranes also allows higher myoglobin removal in patients with rhabdomyolysis.

RRT will naturally be a second line strategy. In our view, the choice of RRT modality will largely depend on the available techniques. The efficacy and tolerance will however largely rely on RRT prescription. Using short high efficiency dialysis (intermittent dialysis) will require high blood and dialysate flow to remove sufficient amount of potassium (e.g., blood flow 250 mL/min and dialysate flow 500 mL/min) allowing rapid drop of serum potassium but with a risk a rebound after stopping RRT (Fig. 4). Clearance of potassium using continuous hemofiltration is proportional to ultrafiltrate rate (Fig. 4). We therefore advise a high ultrafiltration rate at the initiation of the technique (e.g.,  ≥ 45 mL/kg/h) when using this modality. This ultrafiltration rate can be lowered when serum potassium is controlled (e.g., 25 mL/kg/h).

Both techniques expose the patient to the risk of secondary hypokalemia. Importantly, both hyperkalemia and a rapid decrease in serum potassium are associated with cardiac events and sudden death in patients with end-stage kidney disease [81, 82]. Long inter-dialytic periods expose patients to consequences of hyperkalemia and cardiac conduction disorders while intradialytic periods and postdialytic periods are associated to increase cardiac excitability and arrhythmic disorders. Rapid decreases in serum potassium using a potassium dialysate concentration ≤ 2 mmol/L was associated with a doubling of risk of sudden cardiac arrest in a recent study [82]. This arrhythmogenic propensity of RRT is enhanced by simultaneous combined stresses including ischemia (hypovolemia), hypoxia, electrolyte changes (calcium, magnesium, citrate, acetate) and potential removal of cardiac medications. Studies have shown that the frequency of premature ventricular contractions during dialysis is less common when using a dialysate potassium concentration of 2.0–3.0 mmol/L, compared ≤ 2.0 mmol/L [83]. More recently, Ferrey et al. [84] examined the association of dialysate potassium concentration with all-cause mortality risk in chronic hemodialysis patients. They observed that a dialysate potassium concentration of 1 mEq/L was associated with higher mortality compared to higher concentrations. Taken altogether, these data suggest using a potassium dialysate concentration ≥ 2.0 mmol/L to avoid a too rapid drop in serum potassium using dialysis. Treatment of hyperkalemia using peritoneal dialysis has been described anecdotally and appears feasible when alternatives are not readily available [85]. Alternatives to prevent rapid and profound drop of serum potassium is to use low flow techniques (i.e., continuous hemofiltration, continuous hemodialysis or slow low efficiency or extended dialysis) (Fig. 4) once acute severe hyperkalemia has been controlled. Continuous techniques will further largely prevent rebound of serum potassium observed after intermittent dialysis. Finally, extended or continuous session with high flow should be considered in patients with ongoing uncontrolled cause of hyperkalemia (i.e., rhabdomyolysis, tumor lysis syndrome).

### Who should be treated for hyperkalemia?

Even though hyperkalemia has been associated with mortality in different settings [5], the potential side effects of hyperkalemia treatment should not be overlooked. Tailoring treatment to the patient condition and situation might limit the risk of under or over-treating hyperkalemia [34].

**Fig. 5:**
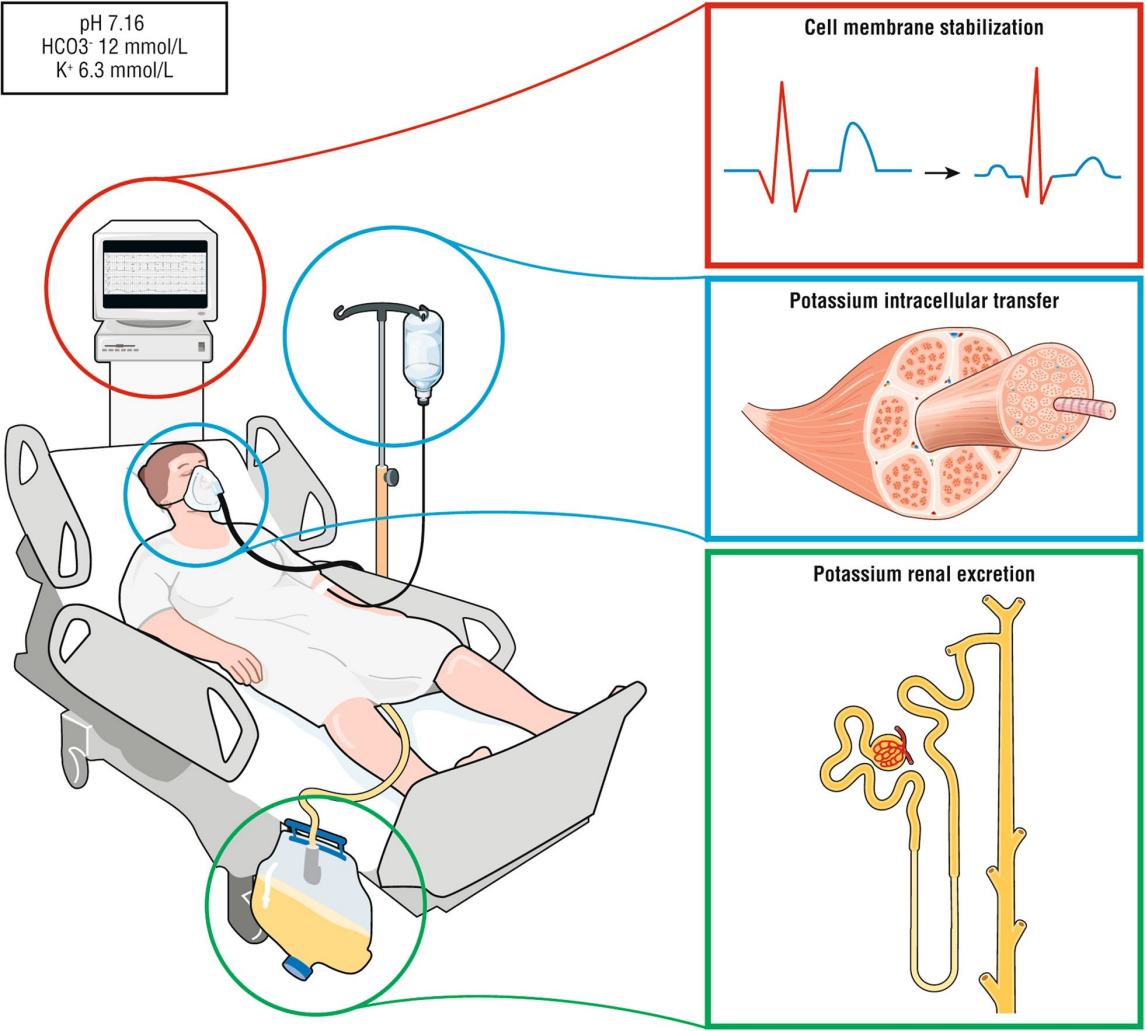
First-line treatment of hyperkalemia. During hyperkalemia with ECG modifications, first-line therapy should consist on cardiomyocyte stabilization using calcium salt or hypertonic sodium (red panel), second line therapy on treatment leading to a fast transfer of potassium from extracellular to intracellular space using either insulin–glucose i.v, aerosol of β2 agonist and/or sodium bicarbonate (in case of metabolic acidosis and hypovolemic patient) depending of the patient’s comorbidities and clinical status. Insulin–glucose is recommended as the first-line treatment in severe hyperkalemia (i.e., above 6.5 mmol/L) but close glucose monitoring is mandatory. β2 agonists can be used in spontaneously breathing patients but with safety concerns in patients with unstable angina or cardiac failure. Hypertonic sodium bicarbonate should probably be restricted to hypovolemic patients with metabolic acidosis (blue panel). Strategies increasing potassium renal excretion decreases the total potassium pool (i.e., hemodynamic optimization and correction of acute kidney injury or loop Henle diuretics in patients with fluid overload) (green panel). Indications of renal replacement therapy are patients with severe acute kidney injury associated to severe hyperkalemia or persistent hyperkalemia despite first-line medical treatment

The evaluation of hyperkalemia should always include assessment for the rapid need of membrane stabilization treatment (i.e., calcium or hypertonic sodium solutions) and should be considered in patients with cardiac conduction or rhythm abnormalities (Figs. 1 and 5). When the clinical scenario and absence of ECG changes do not support the likelihood of hyperkalemia, the potassium measurement should be repeated to exclude factitious hyperkalemia (or pseudo-hyperkalemia). A result of kalemia in delocalized biochemistry (i.e., blood gas analyzer) could probably be used to detect hyperkalaemia and start a treatment in high-risk patients (e.g., patients with severe metabolic acidosis, AKI or CKD).

Efficacy and tolerance of treatment may vary widely according to the clinical scenario (Table 2). Insulin–glucose infusion appears to be appropriate for severe hyperkalemia due to its efficacy and reproducible lowering of serum potassium levels, with close serum glucose monitoring (Fig. 5). However, the impact of this regimen in critically ill patients with insulin resistance or dysglycemia remains unclear. Hypertonic sodium bicarbonate combines fluid loading, cardiac membrane stabilization and serum potassium lowering and is most appropriate in patients with severe metabolic acidosis, AKI and hypovolemia. Aerosolized β-2 agonists are more easily used in spontaneously breathing patients and appear to have similar efficacy to the insulin–dextrose combination in lowering serum potassium. However, the use of β-2 agonists in patients with cardiac hyperexcitability, baseline high sympathetic activity or with unstable coronary disease is potentially associated with severe side effects or decreased efficacy. In addition, efficacy in mechanically ventilated patients is unknown. Serial serum potassium measurements after first-line treatment allow providers to assess the initial response and need for a second line strategy. RRT is usually required in patients with severe AKI with oliguria or anuria who are not expected to rapidly recover (e.g., AKI unresponsive to hemodynamic optimization, unresponsive to diuretics), in patients with end-stage chronic kidney disease admitted for an acute condition and in the setting of severe AKI and hyperkalemia (i.e.,  > 6.5 mmol/L) and in patients with hyperkalemia resistant to medical therapy [8, 34].

Finally, identification and treatment of the cause and contributing factors of hyperkalemia should be performed simultaneously. Identification of the cause of AKI and rapid correction of contributing factors of AKI may allow faster recovery.

## Conclusion

Recognition of hyperkalemia-related ECG changes is central in the choice of strategy to treat the patient. ECG changes should prompt urgent medical intervention including both cardiac protection and potassium-lowering treatment. Tailoring treatment of hyperkalemia to the patient condition and situation will limit the risks of treatments side effects. Efficacy and tolerance remain however poorly explored in acute setting. There is a need for further research to evaluate both efficacy and side effects of different strategies in the acute setting.

## Additional file

**Additional file 1: Figure S1.** Gastrointestinal absorption site of ZS-9, SPS and patiromer. The majority of potassium is in the distal gastrointestinal (GI) tract (e.g., the colon). Both sodium polystyrene sulfonate (SPS) and patiromer are concentration dependent binding (with patiromer being better than SPS). Since there is not relatively much potassium in the early part of the GI tract, SPS and patiromer have less of an effect because there is less for them to bind. Furthermore divalent cation (Ca^2+^ and Mg^2+^) are inadvertently pick up as well. On the contrary, sodium zirconium cyclosilicate (ZS9), which is much more attracted to potassium and more specific than SPS or patiromer (binding coefficient much higher), that it can bind potassium in low concentration environments with less competition with divalent cation, so it starts binding earlier in the GI tract.

## Abbreviations

K^+^: potassium ion
AKI: acute kidney injury
ED: emergency department
ECG: electrocardiographic
SID: strong ion difference
RCT: randomized controlled trial
NKCC: Na–K–Cl cotransporter
SPS: sodium polystyrene sulfonate
US: USA
EU: European Union
ZS-9: sodium zirconium cyclosilicate
RRT: renal replacement therapy
