# Supplementary figures and images for: Management of hyperkalemia in the acutely ill patient
Source: Ann Intensive Care. 2019 Feb 28;9:32. doi: 10.1186/s13613-019-0509-8 (PMC6395464; doi:10.1186/s13613-019-0509-8)

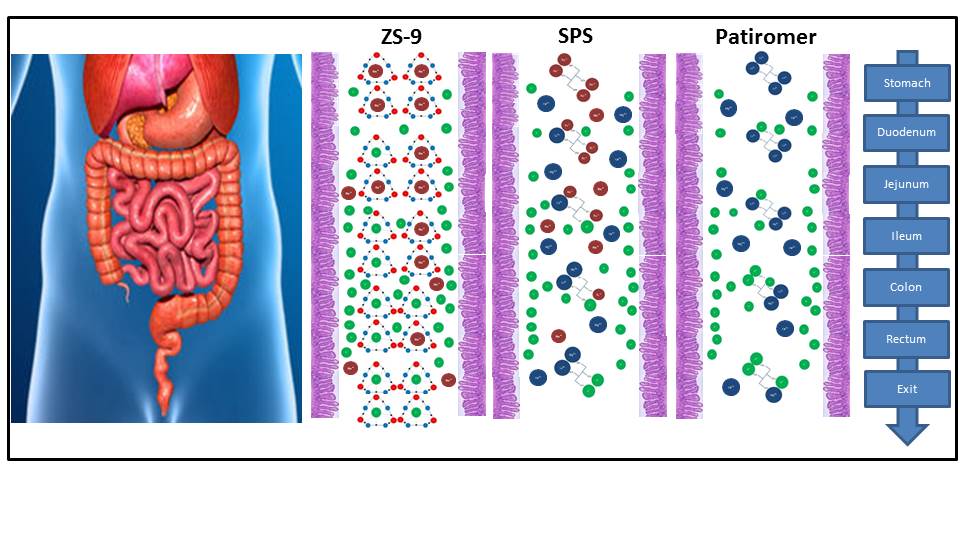

Supplement: Supplementary file 1 — Additional file 1: Figure S1. Gastrointestinal absorption site of ZS-9, SPS and patiromer. The majority of potassium is in the distal gastrointestinal (GI) tract (e.g., the colon). Both sodium polystyrene sulfonate (SPS) and patiromer are concentration dependent binding (with patiromer being better than SPS). Since there is not relatively much potassium in the early part of the GI tract, SPS and patiromer have less of an effect because there is less for them to bind. Furthermore divalent cation (Ca2+ and Mg2+) are inadvertently pick up as well. On the contrary, sodium zirconium cyclosilicate (ZS9), which is much more attracted to potassium and more specific than SPS or patiromer (binding coefficient much higher), that it can bind potassium in low concentration environments with less competition with divalent cation, so it starts binding earlier in the GI tract. [file 13613_2019_509_MOESM1_ESM.jpg]
